# Gendered intergenerational educational mobility patterns converge in the cohort sequence: evidence from Switzerland using administrative data

**DOI:** 10.3389/fsoc.2023.1172553

**Published:** 2023-05-09

**Authors:** Richard Nennstiel, Rolf Becker

**Affiliations:** Department of Sociology of Education, Institute of Educational Science, University of Bern, Bern, Switzerland

**Keywords:** educational mobility, educational expansion, gender differences, relative mobility, absolute mobility, administrative data

## Abstract

In many societies, educational attainment determines social inequality in terms of life chances, and at the same time there is a strong link between social origin and educational success. Therefore, analysis of educational mobility patterns is a central concern for sociologists. In the context of societal changes, such as trend of modernization, educational expansion and significantly increased female participation in education, we use administrative data from different sources (*N* = 556,112) to examine the extent to which absolute and relative intergenerational educational mobility has changed in Switzerland for women and men from the 1951–1990 birth cohorts. We show that there is significantly more upward than downward mobility, while a large proportion of individuals are laterally mobile. By looking at absolute mobility patterns by cohort and gender separately, we extend previous research and show that the decreasing absolute mobility rates are due to the changing educational composition of the parental generations. Following on from previous studies, we reveal that the observed trend toward less relative social mobility continues in the youngest cohorts. It is also worth noting that, while the father's educational attainment has a higher predictive power for children's education in all cohorts, the impact of the mother's education approaches that of the father. Overall, the mobility patterns of men and women converge very strongly over the cohort sequence. Beyond these substantive points, our study demonstrates the potential of using administrative data for social stratification research.

## 1. Introduction

In many societies, there is a strong link between educational attainment and life chances (Müller and Jacob, 2008; Müller and Kogan, 2010; Bukodi et al., 2018; Virdia and Schindler, 2019). This relationship is observable across a range of societal indicators, including income disparities based on educational level (Korber and Oesch, 2019; Alda et al., 2020), disparities in occupational status by educational attainment (Becker and Blossfeld, 2022), elevated levels of unemployment risk among less educated individuals (Neugebauer and Weiss, 2018), differential political engagement (Hillygus, 2005) and variations in partnership behaviors that depend on educational background (Becker and Jann, 2017). The relationship also extends to factors such as health (Leopold and Engelhardt, 2011; Remund and Cullati, 2022) and mortality risk (Unger et al., 2009; Torssander and Erikson, 2010).

Given this multifaceted and central role of educational attainment in shaping an individual's life course, the study of educational inequalities is a major area of research within the discipline of sociology (e.g., Shavit and Blossfeld, 1993; Solga and Becker, 2012). The study of educational inequality occupies an important place in the academic literature (Breen and Jonsson, 2005; Pfeffer, 2008). Research on intergenerational educational inequality specifically assesses the relationship between social background factors such as income, education, occupation and parental social class and their impact on educational opportunities, as well as their persistence and variability over time and across societies (e.g., Shavit and Blossfeld, 1993; Breen and Jonsson, 2005; Becker and Hadjar, 2010; Bukodi and Goldthorpe, 2013; Torche, 2015; Becker and Mayer, 2019; Breen and Müller, 2020).

The analysis of intergenerational educational mobility is dominated by the analytical concepts of absolute and relative mobility (Breen, 2004; Becker, 2006, 2007; Becker and Hadjar, 2010; Torche, 2015). Absolute educational mobility is the direct comparison of the educational attainment of parents and children: how many children achieve a higher, lower or equal level of education than their parents? Relative educational mobility, on the other hand, measures the relationship between the education of parents and of their children: how strongly is parental education related to the education of their children? We examine both absolute and relative intergenerational educational mobility for men and women in the cohort sequence for Switzerland.

Since the mid-20th century, there has been widespread expansion of the educational system in numerous countries around the globe (Breen et al., 2009; Müller and Kogan, 2010; Breen and Müller, 2020), leading to an increase in the length of time spent in education as well as in the attainment of higher educational qualifications. This expansion has been perceived by several authors as particularly advantageous for women, who have managed to reverse their previously unfavorable position within the educational system in comparison to men in various domains (Buchmann et al., 2008; Breen et al., 2010, 2012; DiPrete and Buchmann, 2013; Becker, 2014; Blossfeld et al., 2015).

The educational expansion and the substantial surge in women's educational participation presents several exciting avenues for research on educational mobility. First, it offers an opportunity to assess how this process has altered mobility patterns and educational inequalities based on social background (Shavit and Blossfeld, 1993; Becker and Zangger, 2013; Zangger and Becker, 2016; Blossfeld, 2020). Second, the heightened educational participation of women is of particular interest in the study of mobility patterns, as intergenerational educational mobility research commonly relies on the highest parental educational attainment as a measure of social background (the dominance approach: Torche, 2015; Thaning and Hällsten, 2020), which in older birth cohorts may frequently pertain to the father's education (the conventional approach). Given the significant increase in women's participation in education, the question arises: to what extent does the mother's education influence educational attainment, and thus educational mobility processes? Additionally, the possibility of gender-based patterns of inequality—the extent to which fathers have a greater impact on their sons, and mothers on their daughters—is a topic of great curiosity.

An interesting U-shaped pattern emerges with respect to relative educational mobility in Switzerland. For cohorts born before the 1960's, relative mobility increases and, for cohorts born after, relative mobility decreases again, while the change in relative mobility patterns is more pronounced for women than for men (Jann and Combet, 2012; Jann and Seiler, 2014; Zangger and Becker, 2016; Seiler, 2018). Little is known about absolute educational mobility in Switzerland and its change across birth cohorts, as research on this topic is scarce for this country. As the interrelationship between technological change and education has become stronger in Switzerland across successive birth cohorts (Glauser et al., 2019), one might expect absolute educational mobility to have increased. In addition, the increased demand for highly skilled workers in the labor market is likely to have contributed to an increase in the absolute level of educational mobility across cohorts. Furthermore, due to the tertiarization of employment and the increased returns to higher education, women might have benefited from this societal change and, as a result, may experience higher rates of educational upward mobility compared to men (Kriesi and Leemann, 2020).

Our empirical analysis ties in with existing international studies (Breen et al., 2010, 2012) and attempts to fill several research gaps. It contributes to the state of research on intergenerational educational mobility, first by presenting analyses of absolute educational mobility in the cohort sequence for Switzerland, and second by examining paternal and maternal education individually (in addition to the dominance approach) to see if gender-specific mobility patterns can be identified for absolute and relative educational mobility patterns. In this respect, our contribution is an explorative application of the conventional approach, the dominance approach and the joint approach in the analysis of intergenerational educational mobility (Torche, 2015). Third, by examining the 1950–1990 birth cohorts, we can also draw conclusions about the youngest birth cohorts in Switzerland, extending previous studies in terms of continued observation of the output of the Swiss educational system (Jann and Combet, 2012; Becker and Zangger, 2013; Jann and Seiler, 2014; Zangger and Becker, 2016; Seiler, 2018). In addition to these substantive contributions to the state of research, our methodological approach represents an innovative method in intergenerational educational mobility research (Wanner, 2022). Unlike many previous studies, we do not use survey data; rather, we analyze administrative data from different data sources (censuses, structural surveys and Population and Households Statistics [STATPOP]). Linking these datasets allows us to obtain an analytic sample with case numbers that significantly exceed those of previous studies (*N* > 500,000), enabling us to calculate detailed subgroup analyses by birth cohort, gender and parental educational background. In addition, the data allow us to identify parents and children who no longer live in the same household. This enables us to draw more generalizable conclusions than previous studies using administrative data (e.g., Bauer and Riphahn, 2007). Furthermore, we are able to obtain direct educational information from parents without having to rely on information from their children, as is often the case in survey data (Breen and Jonsson, 1997; Hovestadt and Schneider, 2021). In addition, several biases regarding willingness to participate and response behavior are known to arise from survey research (Groves et al., 2011; Dillman et al., 2014). Since participation in the census and structural survey is mandatory, these biases are expected to be smaller than in classical surveys.

In the next section, we present the state of research on intergenerational educational mobility in Switzerland. After this, we present our theoretical considerations. We then describe our data sources and how we linked them, the selection of our analytic sample and the operationalization of the variables used in the analyses. Subsequently, we present our results, before concluding with a discussion of our findings in light of the state of research.

## 2. State of research

As mentioned above, there is a lack of research on absolute intergenerational educational mobility in Switzerland. Levy et al. (1997) presented an analysis based on survey data from 1991 (*N* = 1,869). They were able to show the following educational mobility for this group of people: 40% had the same education as their parents and 43% had attained a higher level of education, while 17% had attained a lower level of education. Using 2000 census data, Bauer and Riphahn (2007) examined the educational mobility of 17-year-olds still living in the same household as their parents (*N* = 74,147). They found that, among natives for whom educational information was available both for the children and for the father, 65% were laterally mobile (i.e., had a level of education equivalent to that of their parents), 25% were upwardly mobile, and 10% were downwardly mobile. With respect to the mother's education, 57% were laterally mobile, 36% were upwardly mobile and 7% were downwardly mobile. The studies mentioned above have in common that they show a low degree of educational downward mobility for Switzerland. In addition, they reveal a high degree of lateral mobility and that there is significantly more upward mobility than downward mobility. These studies do not provide information on how these mobility patterns vary by gender or by birth cohort.

Recently, an increasing number of studies have been published on relative educational mobility. Many studies also examine the relationship between parental social background (measured by education, class or status, or a combination of these) and the education of their children, or specific educational transitions in the educational trajectory (e.g., Bauer and Riphahn, 2007; Hadjar and Berger, 2010; Jann and Combet, 2012; Becker and Zangger, 2013; Zangger and Becker, 2016; Falcon, 2020). In presenting the state of the research on relative educational mobility, we focus only on studies that examine the relationship between parental education and the highest educational attainment of children in general.^1^

Buchmann et al. (1993) investigated the impact of paternal years of education (and parental status) on years of education for men and women in the 1950 and 1960 birth cohorts (*N* = 1,732). Their findings suggest that relative mobility remained constant across these two birth cohorts. Based on a survey from 1991 (*N* = 1,310) and a second survey from 1999 (*N* = 1,632), Bergman et al. (2002) examined relative educational mobility separately by gender, parental education, and survey year. Comparing the two surveys, a decrease in relative mobility over time was detected. However, the authors pointed out that this result might also have been due to a change in the classification of education across the different years. They also found that the father's education had a greater impact on the child's educational attainment than the mother's education. Joye et al. (2003) used different surveys from 1975, 1991, and 1999 to analyze the relative educational mobility of sons (aged over 35) and fathers. They distinguished two age cohorts (aged 35–49 and 50–65) in each survey year and estimated educational mobility parameters separately. They found that, especially in 1975 and 1991, the younger cohort had higher relative mobility than the older cohort. In 1999, the mobility patterns of the two age cohorts converged. The older cohort became slightly more mobile between 1975 and 1991, and the younger cohort became less mobile between 1991 and 1999. These results suggested that relative mobility patterns between birth cohorts first increased, and then stagnated or slightly declined.

A study by Pfeffer (2008), which included the educational mobility patterns of more than 20 countries, also reported relative mobility patterns for Switzerland. Based on survey data from the 1990s (*N* = 946), four age cohorts were differentiated (aged 26–35, 36–45, 46–55, and 56–65) and their relative mobility parameters were estimated. According to these results, relative mobility was very stable across the age cohorts and there was virtually no change in relative mobility patterns. In a comparison of the countries studied, Switzerland had the fourth lowest relative mobility rate. Only Germany, Belgium and Slovenia had lower relative mobility rates.

Jann and Combet (2012) examined relative educational mobility in Switzerland using a cumulated dataset of several surveys (*N* = 25,858). They classified 30–69-year-olds into five different birth cohorts (1891–1941, 1942–1949, 1950–1957, 1958–1964, and 1965–1980) and calculated the relationship between parental education and children's education, controlling for parental social class separately by gender and birth cohort. For men, they found an increase in relative intergenerational mobility between the 1945 and 1960 cohorts. For women, the process of improvement in relative mobility began in earlier birth cohorts and was more pronounced than for men. In the most recent cohort in the study (born around 1970), a decline in relative social mobility could be observed for both men and women. This inverted U-shaped relationship was more obvious for women than for men. Jann and Seiler (2014) also examined the relative educational mobility of 30–69-year-olds in Switzerland, employing a combination of different surveys (*N* = 33,068). They examined the 1922–1982 birth cohorts, which they divided into five cohorts spanning 6–16 birth years. They also found a U-shaped pattern of relative mobility (reflecting the impact of the highest educational attainment of parents on the education of their children) for both genders. Relative educational mobility increased up to birth cohorts born around 1960, with relatively stable relative mobility patterns for birth cohorts born from 1950 to the mid-1960s. For birth cohorts born from the mid-1960s onward, a deterioration in relative mobility patterns was observed. Considering various control variables, they found that the U-shaped relationship was more pronounced for women than for men. For both genders, however, a deterioration in relative mobility patterns could be observed for birth cohorts from the mid-1960's onward.

Although the findings of the research on relative educational mobility in Switzerland are not beyond dispute, the following conclusions can be drawn from the studies mentioned above. Most studies report stable or improving patterns of relative mobility for cohorts born between 1920 and 1960. For cohorts born from the mid-1960s onward, the results suggest that relative mobility patterns are deteriorating again, and that parental education is once again becoming a stronger determinant of children's education. A pattern of increasing relative mobility for the 1940s and 1950s birth cohorts, followed by stagnating or diminishing mobility rates for subsequent cohorts, has also been observed in other countries (see, for example, Barone, 2019). Regarding gender differences in relative mobility patterns, the amplitude of change over time is greater for women than for men. There is no clear evidence on whether the education of the same-sex parent has a stronger influence on children's mobility patterns. In general, it seems that the education of the father has a stronger influence on the education of the child than the education of the mother (e.g., Bergman et al., 2002).

## 3. Theoretical considerations

Compared to other countries, the educational expansion in Switzerland started late in the post-war period (Buchmann et al., 1993), and has followed a hesitant course since the 1970's (Becker and Zangger, 2013). However, due to the expansion of the educational system since the 1990's, it might be expected that younger birth cohorts have better opportunities for educational mobility (Glauser et al., 2019). Additionally, there is empirical evidence of the increased influence of educational attainment on occupational status across birth cohorts (Becker and Blossfeld, 2022). This may indicate that across birth cohorts higher educational attainment is necessary for children to maintain the occupational status of their parents and the related class position. Therefore, we suppose that there has been an increase in educational upward mobility across birth cohorts.

Hypothesis 1: Absolute educational mobility increases across cohorts.

Although the expansion of education in Switzerland has augmented opportunities for educational mobility (Glauser et al., 2019), this does not implicitly suggest that the correlation between parental education and children's education will attenuate over time. On the contrary, we anticipate the influence of parental education on their children's education will intensify, particularly as a consequence of the aforementioned developments concerning occupational upgrading and the enhanced linkage between educational attainment and occupational status, as progressively higher educational attainment is necessary to maintain status in the generational sequence (Breen and Goldthorpe, 1997; Becker, 2003). In accordance with prior research (e.g., Jann and Combet, 2012; Jann and Seiler, 2014; Seiler, 2018), we suppose that relative educational mobility across cohorts will diminish.

Hypothesis 2: Relative educational mobility declines across cohorts.

The following theoretical considerations can be made regarding gender-specific changes in educational mobility. Following the argument of detraditionalization, one might assume that educational inequalities between the sexes will have converged. Breen and Goldthorpe (1997) stated that the reason for this is that women in the earlier birth cohorts were more likely to have maintained their intergenerational status through marriage than through gainful employment. According to this, origin-related educational inequalities are expected to have been lower among women than among men. For women in the younger birth cohorts, education and the employment that builds on it are likely to have become more important, so educational disparities among women are expected to have approached those of men. This suggests that, in older cohorts, women display higher relative mobility than men, and that relative mobility patterns equalize in the cohort sequence. Furthermore, absolute mobility patterns are also expected to converge. This will be reflected in improving upward mobility rates for women in the cohort sequence. Women are considered to have been the winners of the educational expansion (Buchmann et al., 2008; DiPrete and Buchmann, 2013), and international studies show that their mobility chances have improved as a result (Breen et al., 2010). For men, the increase in upward mobility rates is expected to be less pronounced; while they have also benefited from the expansion of the education system, the reasons for their educational behavior (e.g., to utilize educational qualifications on the labor market to match the status of their parents) have hardly changed over cohorts.

Hypothesis 3: Educational mobility patterns (absolute and relative) equalize across cohorts between the genders.

## 4. Materials and methods

### 4.1. Data

We use several data sources for our analyses. First, we use data from the 1970, 1980, 1990 and 2000 censuses [Federal Statistical Office (FSO), 2017]. Each census surveyed the entire population. The last census took place in Switzerland in 2000, when the survey system changed. Second, we use data from the five-year cumulative structural surveys from 2011–2015 and 2016–2020 [Federal Statistical Office (FSO), 2021]. Since 2010, a structural survey has been conducted every year, in which a sample of at least 200,000 people over the age of 15 living in private households has been interviewed [Federal Statistical Office (FSO), 2021]. To reduce the length of the structural survey interview, additional information on respondents has also been obtained from other registers (population registers, federal population registers and housing registers). In addition to information about the target person, information about household members is also collected as part of the structural survey. Both the census and the structural surveys are subject to mandatory participation.

Third, we use data from STATPOP [Federal Statistical Office (FSO), 2022], an annual register of the permanent resident population. These data include information on date of birth, gender, place of residence, place of birth and marital status. For our analyses, the FSO has also assigned to these data the ID of the father and mother of each person, if they were identifiable in the registers. We use information from STATPOP for 2010–2020. STATPOP is available from 2010 onward, so it is not possible to match parents and children by ID for the census data. Therefore, we do not use the census data for our mobility analysis, but rather to show univariate distributions of education over time.

**Figure 1:**
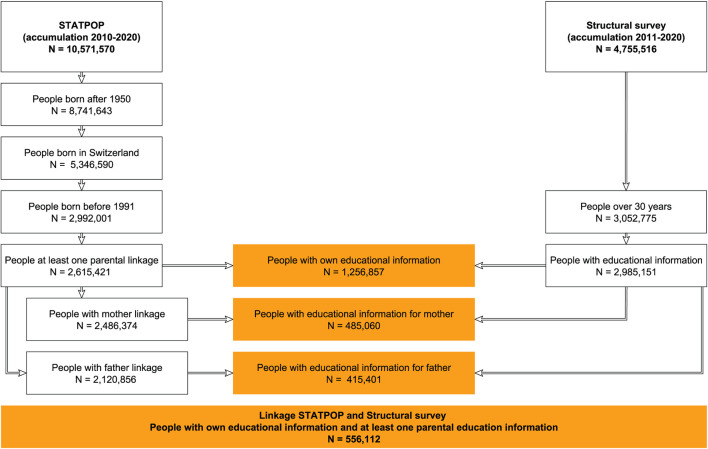
Graphical illustration of the linkage between the datasets. Source: our own depiction.

Our analyses of educational mobility require information from individuals and from their parents. The structural survey asks individuals about their highest level of education, but not directly about the education of their parents or children. Therefore, we have used the IDs provided by the FSO to match individuals to their father and/or mother. To achieve this, we proceeded step by step (see Figure 1).

First, we cumulated data from the 2011–2015 and 2016–2020 structural surveys to create a dataset with N = 4,755,516 unique individuals. We then excluded all individuals who were under the age of 30 at the time of the structural survey.^2^ We imposed this restriction to ensure that the educational processes of the respondents and their parents were complete at the time of measurement (e.g., Jann and Combet, 2012). This left *N* = 3,052,775 individuals in the dataset, for whom we had information on the educational attainment of *N* = 2,985,151.

In a second step, we cumulated the information from STATPOP over the years 2010–2020 to generate a dataset with *N* = 10,571,570 distinct individuals. We know from previous studies that the probability of linkage between parents and children varies with the year and place of birth of the child (Wanner, 2022; see also Supplementary Figure 1 in the Supplementary material). This is because, for parents and children to be linked, both must be listed in the civil registry for at least one year beginning in the late 1990's. For parents of persons born before 1950, the linkage is methodologically unacceptable (<60%) due to age-related mortality. For persons born abroad, the poorer linkage (below 30%) is because their parents were often never registered in Switzerland. For these reasons, we decided to include only individuals born in Switzerland in 1951 or later.^3^ In line with the chosen age limit of 30 years in the structural survey data, we also excluded all individuals born after 1990. Of these *N* = 2,992,001 individuals, *N* = 2,615,421 could be assigned at least one parent, *N* = 2,486,374 could be assigned a mother and *N* = 2,120,856 could be assigned a father.

We then matched the data from the structural survey to STATPOP using the person identifiers (person, person's mother, person's father). In order for a person to end up in our dataset, the person must have had at least one parent assignable in STATPOP (in addition to the age and birthplace restrictions); the person must have been included in the structural survey (either as a target person or as the household member of a target person); and the person must have had a valid education entry. For a parent to end up in our sample, there must have been at least one child in our sample and the parent must have been included in the structural survey (either as a target person or as a household member of a target person), and there must have been a valid education entry. Thus, after linkage, there was educational information for *N* = 1,256,857 individuals. For *N* = 485,060 individuals, we had educational information available for the mother, and educational information was available for the father for *N* = 415,401 individuals. Finally, for *N* = 556,112, there was educational information for the individual and for at least one parent.

### 4.2. Operationalization

Education is measured for both parents and children through their highest educational attainment on a six-level scale. We use the categories provided by the FSO. The following educational qualifications are distinguished: without degree (without an education or vocational training degree [1]), compulsory education (2), vocational education and training (VET [3]), general education schools (baccalaureate – e.g., university admission degree – and upper secondary specialized schools [4]), professional education and training (PET [5]) and university (universities, universities of teacher education, universities of applied sciences [6]).

To analyze the change in mobility patterns over time, we categorized birth years according to eight five-year birth cohorts: 1951–1955 (1), 1956–1960 (2), 1961–1965 (3), 1966–1970 (4), 1971–1975 (5), 1976–1980 (6), 1981–1985 (7) and 1986–1990 (8). It has to be emphasized that this definition of a cohort is not based on a theory. Rather, it is intended to be linked to existing studies in a pragmatic way (e.g., Hadjar and Berger, 2010; Jann and Combet, 2012). In general, this cohort approach is used because social change in educational behavior occurs across successive cohorts (Becker and Mayer, 2019), as they are the cultural bearers of change in economic, political and cultural orders and social contexts (Ryder, 1965, 1985).

### 4.3. Methods

We measure absolute educational mobility as follows: a person is upwardly mobile if he or she has a higher educational attainment than his or her parents; a person is laterally mobile if he or she has an educational attainment equivalent to that of his or her parents; and a person is downwardly mobile if he or she has a lower educational attainment than his or her parents. We calculate the rates of absolute mobility using cross-tabulations and then plot them using bar charts^4^ and Sankey plots.^5^

We use so-called error reduction measures (PRE) as a measure of relative mobility (Jann and Combet, 2012; Jann and Seiler, 2014).^6^ These PRE measures are advantageous in contrast to the conventional UNIDIFF parameters since they have a substantive interpretation and are comparable across different models (Jann and Seiler, 2014; p. 27). Moreover, the interpretation of these measures is quite intuitive: what do we learn about children's education when we have information about their parents' education? In other words, how much does our prediction error about children's education decrease when we can account for their parents' education? Since we only estimate models with one independent variable (the parents' highest educational attainment or the father's education or the mother's education), we use the Mc Fadden's pseudo-R^2^ (McFadden, 1974) calculated from the multiple logistic regression for children's education as a measure of relative mobility (Jann and Seiler, 2014; p. 10). Mc Fadden's pseudo-R^2^ is calculated using the following formula:


(1) $$\begin{array}{l} R_{MF}^{2}=1-\frac{LL_{1}}{LL_{0}} \end{array}$$

The pseudo-R^2^ employed in our analysis is computed as 1 minus the ratio of the log-likelihood of a model incorporating parental education as a predictor (*LL*_1_) to the log-likelihood of a model without explanatory variables (*LL*_0_). This pseudo-R^2^ can assume values between 0 and 1. The pseudo-R^2^ can be interpreted as an indicator of the improvement in model fit (measured using log-likelihoods) when comparing a model with explanatory variables (e.g., parental education) to a model without explanatory variables (Hemmert et al., 2018). Elevated pseudo-R^2^ values signify enhanced model fit when comparing the model incorporating explanatory variables to the model devoid of such variables. In our application example, higher pseudo-R^2^ values correspond to lower relative mobility.

We estimate multinomial logistic regressions separately for each cohort. As a first step, we use the highest parental education as an independent variable and run the models for all individuals, as well as separately by gender. In further steps, we use the father's education and the mother's education as independent variables.^7^

## 5. Results

### 5.1. Univariate distributions

**Figure 2:**
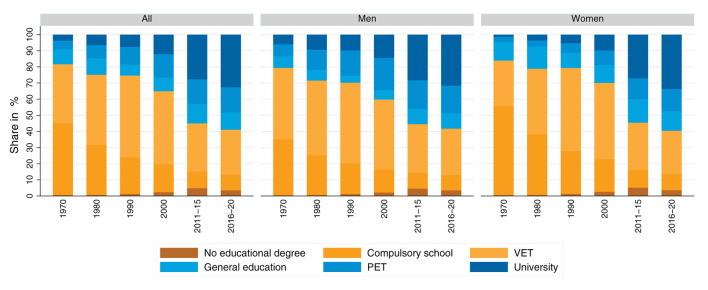
Highest educational attainment of 30–39-year-olds, separated by gender and year of data collection. Note: For this chart, we use only the sample of target persons from the structural surveys. We used the weights provided by the FSO and first calculated the shares for each of the individual years of the structural surveys, and then averaged them over five years. N_1970, Men_ = 413,132, N_1970, Women_ = 393,454; N_1970, Total_ = 806,586. N_1980, Men_ = 477,515, N_1980, Women_ = 461,463; N_1980, Total_ = 938,978. N_1990, Men_ = 532,490, N_1990, Women_ = 507,938; N_1990, Total_ = 1,040,428. N_2000, Men_ = 556,404, N_2000, Women_ = 559,765; N_2000, Total_ = 1,116,169. N_2011−15, Men_ = 92,193, N_2011−15, Women_ = 99,531; N_2011−15, Total_ = 191,724. N_2016−20, Men_ = 93,809, N_2016−20, Women_ = 100,803; N_2016−20, Total_ = 194,612. In 1970 and 1980, the percentage of people who had no educational degree was less than 2%, so this group is not visible in the graphs for these years. Source: Census 1970–2000, and structural survey cumulations 2011–2015 and 2016–2020; our own calculations.

Figure 2 depicts the expansion of education in Switzerland. The share of individuals with no more than compulsory schooling has declined over time, while the share of individuals with tertiary education (PET and university) has increased significantly. With regard to gender differences, it can be seen that women have been able to reduce their disadvantages in terms of educational attainment and that they now have slightly higher rates of university education than men.

**Figure 3:**
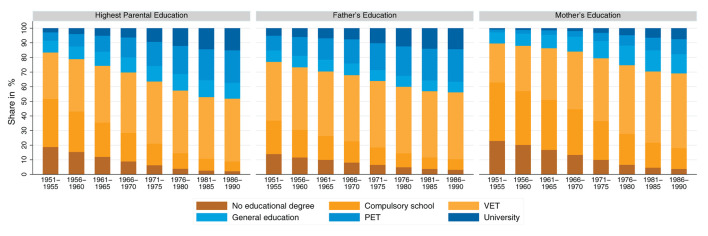
Highest parental educational attainment, separated by parental education and birth cohort. Source: Structural survey cumulations 2011–2015 and 2016–2020, and STATPOP 2010–2020; unweighted, our own calculations.

For intergenerational mobility processes, not only the education of individuals is important, but also the distribution of parental educational attainment (see Figure 3). It can be seen that the parents of the children of the 1951–1990 birth cohorts also show a trend toward higher educational attainment. Comparing the educational attainment of fathers with that of mothers shows that fathers have higher educational attainment than mothers.^8^ Although the advantages of fathers diminish slightly in the cohort sequence, they are still clearly discernible even for the youngest cohort considered here.

### 5.2. Absolute mobility patterns

**Figure 4:**
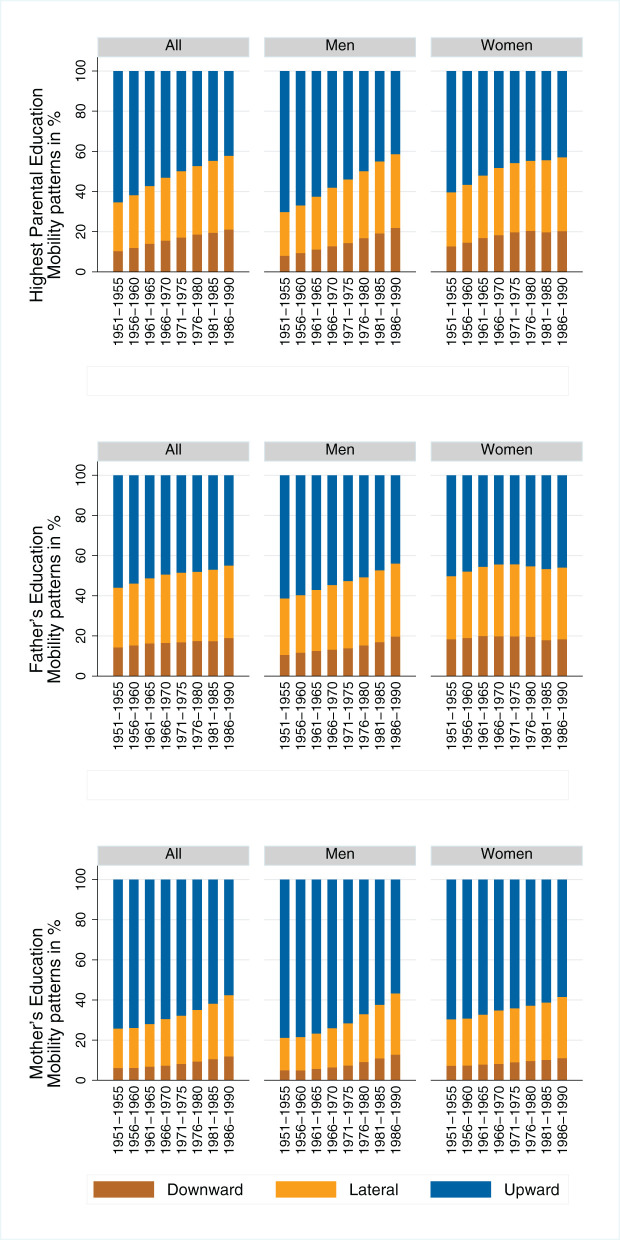
Absolute mobility patterns across birth cohorts, separated by gender and operationalization of parental education. Source: Structural survey cumulations 2011–2015 and 2016–2020, and STATPOP 2010–2020; unweighted, our own calculations.

Figure 4 shows the patterns of absolute mobility across birth cohorts. There is a trend toward less upward mobility, more downward mobility and increased lateral mobility. The absolute mobility patterns are similar for men and women. It is noticeable that, when absolute mobility is calculated using the mother's education, more upward mobility and less downward mobility is detected than when absolute mobility is measured using the father's education. However, the time trends observed for absolute mobility are very similar, regardless of the choice of parental education reference. In a single case—when women are compared with their fathers—there are virtually no changes over time. Overall, for both men and women, even among the youngest cohorts, more than 40% still achieve a higher level of education than their parents, and 80% achieve at least the same level of education as their parents.

**Figure 5:**
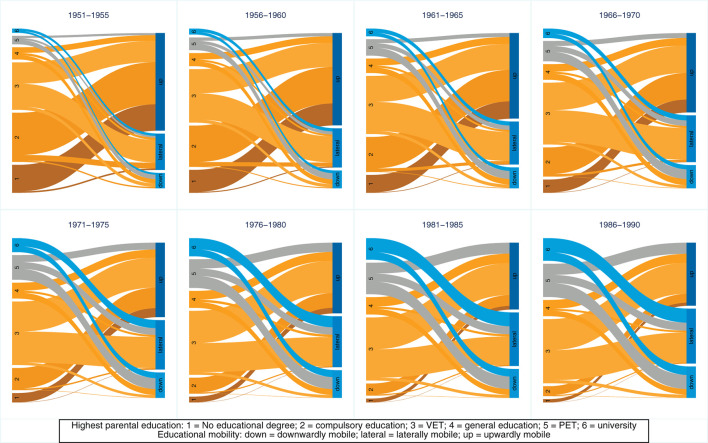
Sankey plot of educational origin and educational absolute mobility (measured using highest parental education), by birth cohort. Source: Structural survey cumulations 2011–2015 and 2016–2020, and STATPOP 2010–2020; unweighted, our own calculations.

This trend toward increased lateral mobility and the decline in upward mobility may be based on the fact that the composition of parents' educational attainment has changed significantly during the course of educational expansion (see Figure 3 for similar reasoning regarding intergenerational class mobility in Great Britain and educational downward mobility in Germany: see Goldthorpe, 2016 and Blossfeld, 2022). The more parents attain higher- and highest-level educational degrees, the more their children are at risk of downward mobility. Furthermore, when calculating rates of absolute mobility, ceiling and floor effects can occur. To give an example for these ceiling and floor effects: children whose parents attain a university degree cannot be upwardly mobile (ceiling effect), and children whose parents have no educational degree cannot be downwardly mobile (floor effect). Figure 5 shows a Sankey plot of the relationship between children's educational mobility and their parents' highest educational attainment (for all individuals, undifferentiated by gender). It is striking that the groups of origin with no educational degree and compulsory schooling, from which a large proportion of children are upwardly mobile, become significantly smaller over the cohorts. It is also noticeable that the origin groups with general education and above, from which a large proportion of children are downwardly mobile, become proportionally significantly larger. These shifts could explain why upward mobility decreases over the cohort sequence, and why lateral and downward mobility increases.

**Figure 6:**
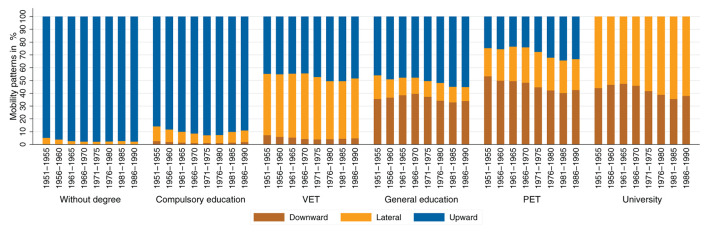
Absolute mobility patterns conditional on parental education (measured using highest parental education), by birth cohort. Source: Structural survey cumulations 2011–2015 and 2016–2020, and STATPOP 2010–2020; unweighted, our own calculations.

**Figure 7:**
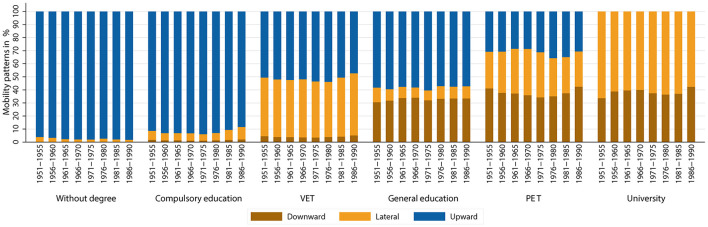
Absolute mobility patterns conditional on parental education (measured using highest parental education), by birth cohort (men only). Source: Structural survey cumulations 2011–2015 and 2016–2020, and STATPOP 2010–2020; unweighted, our own calculations.

**Figure 8:**
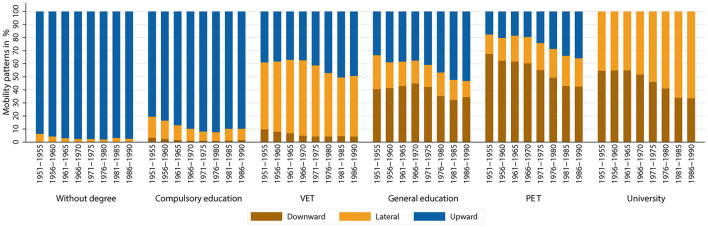
Absolute mobility patterns conditional on parental education (measured using highest parental education), by birth cohort (women only). Source: Structural survey cumulations 2011–2015 and 2016–2020, and STATPOP 2010–2020; unweighted, our own calculations.

Looking at the absolute patterns of educational mobility conditional for the group of origin of individuals across cohorts, an interesting pattern emerges (see Figure 6). In the two lowest educational groups, there are hardly any changes across birth cohorts. For the other educational groups, however, absolute mobility patterns tend to stagnate or worsen up to the birth cohorts of the 1960's. For the cohorts born after 1970, the share of upward and lateral mobility increases, and the share of downward mobility decreases within the groups. This suggests a slight improvement in absolute mobility patterns. The patterns seen in Figure 4 therefore appear to be due to the changing educational composition of parents rather than to diminishing absolute mobility patterns. Looking at the educational mobility patterns conditional on group of origin separately by gender (see Figures 7, 8), interesting differences emerge. In the older birth cohorts, the conditional mobility patterns of men and women differ, especially in the higher groups of origin (at least in terms of general education). Women are more often downwardly mobile and less often upwardly or laterally mobile than men. While there are no substantial changes in absolute mobility patterns for men in any of the origin groups, there is a change in the mobility patterns for women in all origin groups. Upward mobility increases in the cohort sequence, and downward mobility decreases at the same time. This process of improving conditional mobility patterns for women is particularly evident for cohorts born from the 1970's onward. Over time, the mobility patterns conditional on parental education have converged so that hardly any differences can be observed in the most recent cohorts. If anything, the absolute mobility patterns of women are now slightly better than those of men.

### 5.3. Relative mobility patterns

**Figure 9:**
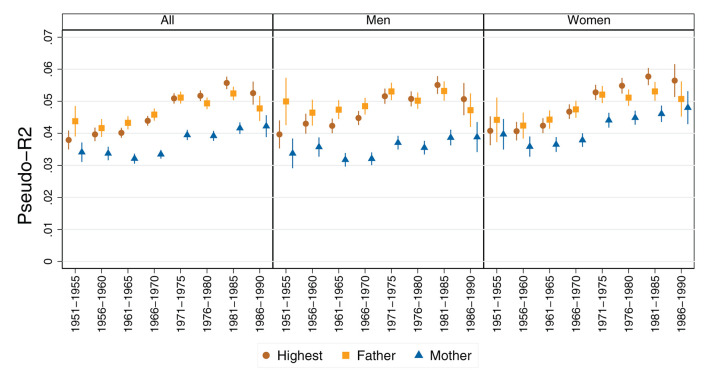
Relative mobility patterns (pseudo-R^2^) by gender across birth cohorts, differentiated by operationalization of parental education. Source: Structural survey cumulations 2011–2015 and 2016–2020, and STATPOP 2010–2020; unweighted, our own calculations. Note: Estimates based on 1,000 bootstrap replications of multinomial logit models.

Figure 9 shows relative mobility patterns (measured using pseudo-R^2^) across birth cohorts and different operationalizations of parental education. For the cohorts born between 1950 and 1965, relative mobility has remained stable regardless of the reference point of parental education.^9^ The predictive power of parental education for children's educational attainment has remained stable. For cohorts born from the mid-1960s onward, a decline in relative mobility is evident. The predictive power of parental education for children's education increases. Interestingly, these patterns are observed regardless of the operationalization of parental education (the parents' highest educational attainment, the father's education or the mother's education). The relative patterns of mobility between the sexes differ slightly only in the oldest cohorts, such that the predictive power of parental education is slightly stronger for men than for women in these cohorts. For cohorts born in 1965 or later, the relative mobility patterns are very similar: relative mobility is declining for both men and women. It is interesting to note that, for the cohorts born from 1970 onward, maternal education has a slightly greater impact on relative mobility for women than for men. In general, however, we detect that the predictive power of maternal education is lower than that of paternal education or of highest parental education.

Even if a slight deterioration in relative mobility across cohorts is revealed, further interpretation must consider that the pseudo-R^2^ has increased from 0.04 to a maximum of 0.055. In relative terms, this is a significant increase. In absolute terms, however, it is rather small, suggesting that the effect of parental education on children's education has not changed substantially.

## 6. Discussion

The aim of our contribution was to investigate absolute and relative patterns of educational mobility across birth cohorts for Switzerland. Special attention was paid to the detection of gender differences in mobility patterns. We were also interested in calculating these, not only by using the dominance approach (based on the parents' highest educational attainment) or the conventional approach (based on the father's highest education) – which are predominant in mobility analyses – but also by considering the mother's education. Furthermore, we were interested in the extent to which these patterns have changed across birth cohorts over the course of educational expansion.

To map mobility patterns, we combined various administrative data into a dataset of *N* = 556,112 observations. This approach allowed us to analyze a much larger sample than previous studies on intergenerational educational mobility in Switzerland (e.g., Buchmann et al., 1993; Levy et al., 1997; Bergman et al., 2002; Joye et al., 2003; Pfeffer, 2008; Jann and Combet, 2012; Jann and Seiler, 2014; Seiler, 2018). In addition, the use of this data offered further advantages, such as the identification of parents and children living in separate households, allowing for more generalizable conclusions than previous studies based on administrative data (e.g., Bauer and Riphahn, 2007). Moreover, participation in the census and structural survey is mandatory and so parental education information could be obtained without relying on child reports, reducing biases associated with survey participation and response patterns (Breen and Jonsson, 1997; Groves et al., 2011; Dillman et al., 2014; Hovestadt and Schneider, 2021). By using structural survey data covering the period up to 2020, we were able to show that, since 2000, the expansion of higher educational attainment in Switzerland has gained significant momentum, and an ever-larger share of the population has been attaining higher educational qualifications. In line with previous research (e.g., Jann and Combet, 2012; Becker and Zangger, 2013; Zangger and Becker, 2016), we found that there was only a moderate expansion in higher educational attainment up to the year 2000 in Switzerland. As in other industrialized countries, it is evident that women in Switzerland have been able to reduce their previous disadvantages in terms of educational attainment and that they now achieve, on average, higher qualifications than men (Buchmann et al., 2008; DiPrete and Buchmann, 2013). This significant increase in educational attainment after 2000 has not yet been reflected in our analyses of intergenerational mobility, but these developments suggest that mobility patterns in Switzerland may change in future.

As is commonly done in other studies of intergenerational mobility (e.g., Jann and Seiler, 2014), we also analyzed the distribution of origin (parents' educational attainment) across birth cohorts. There, too, we revealed a trend toward an increased share of parents with higher educational attainment (e.g., Jann and Seiler, 2014; Ziefle, 2017; Blossfeld, 2022). It is interesting to note that, on average, fathers continue to have higher educational attainment than mothers, although these differences have narrowed somewhat over time. This trend suggests that there may have been a slight degree of detraditionalization in the educational attainment of the parental generation. These findings are in line with other studies from the Swiss context that examine educational homogamy in couple relationships (Becker and Jann, 2017), concluding that women are significantly more likely to choose partners with an educational attainment higher than their own compared to men.

We supposed that the development of absolute mobility patterns would improve across cohorts and that there would be more upward mobility. We expected this because, on the one hand, educational opportunities have improved over the course of educational expansion and, on the other, educational labor requirements have increased as a result of the tertiarization of the labor market (Glauser et al., 2019). In line with the detraditionalization thesis (Breen and Goldthorpe, 1997), which posits that women's educational participation will converge with men's as women increasingly realize their status attainment through the labor market and less through the marriage market, we supposed that women in particular are the drivers of increased upward mobility. Consistent with previous studies (e.g., Levy et al., 1997; Bauer and Riphahn, 2007), we found significantly more upward than downward mobility in all cohorts and that a large proportion of mobility is lateral. Contrary to Hypothesis 1, the results of our analyses also showed that mobility outcomes for both men and women have not improved but in fact slightly worsened. Furthermore, mobility patterns between the sexes have become more equal across birth cohorts. Interestingly, the patterns of these temporal trends do not vary much depending on what level of education is used as a reference (the highest level of parental education, the father's education or the mother's education). Even if there is a trend toward more downward and less upward mobility, it is worth noting that, even among the youngest cohorts, 80% of respondents still have at least the same level of educational attainment as their parents.

We have suggested that the deterioration in absolute mobility patterns is due to compositional effects (e.g., Goldthorpe, 2016; Nennstiel, 2021; Blossfeld, 2022). The Sankey plots show how large the shifts in groups of origin have been across cohorts: fewer and fewer children have parents with lower levels of education, and more and more children have parents with higher levels of education. As a result, upward mobility has become increasingly difficult or impossible for a larger share of birth cohorts (e.g., ceiling effects). At the same time, the share of those exposed to the risk of downward mobility has increased. If we also look at absolute mobility rates conditional on parents' educational attainment, we see that they have tended to improve slightly. The share of individuals who were laterally or upwardly mobile among those whose parents had the same educational attainment has increased over the cohort sequence. Looking at this development by gender, it is striking that women have been the main drivers of this development. The conditional absolute mobility rates of men have remained fairly stable across all educational groups. For women, however, conditional upward mobility rates have increased, while downward mobility rates decreased, especially for cohorts born after 1970. Considering the changing educational composition of parents, the results support our expectations that upward mobility rates have increased and that women's mobility in particular has been responsible for this development.

We expected the change in relative mobility patterns to worsen slightly in line with the state of research, continuing the U-shaped relationship found in previous studies (Jann and Combet, 2012; Jann and Seiler, 2014; Seiler, 2018). In line with the detraditionalization thesis (Breen and Goldthorpe, 1997), we expected that relative mobility patterns between the genders would equalize over the cohort sequence and that women would have higher relative mobility than men in the older cohorts. In line with Hypothesis 2, our results suggested that relative mobility patterns initially remained stable across cohorts, and that relative mobility then declined. This is consistent with previous research, which found an inverted U-shaped pattern for birth cohorts born up to the 1970s. We found that the decline in relative mobility has continued and stabilized in subsequent cohorts (those born in the 1980s). The pattern of relative mobility has worsened, and this change is statistically significant. However, in terms of magnitude, this change is rather small. The pseudo-R^2^ of the predictive power of parental education on children's education increased slightly from 0.04 to 0.055 in the cohort sequence. Comparing the different measurement approaches (Torche, 2015), it appears that all three approaches show similar time trends, albeit with different magnitudes. Interestingly, the mother's education generally has less predictive power than the father's education or the parents' highest educational attainment, and this is true for both men and women. The predictive power of the father's highest educational attainment and of the highest parental educational attainment are similar (see also the additional analyses in Supplementary Figure 11 in the Supplementary material). This finding suggests that the dominance approach is still relevant in the Swiss context. It is interesting to note that the predictive power of maternal education converged with the other two approaches across cohorts. In particular, the youngest cohort showed a strong convergence in the predictive power of the different measurement approaches. This could be an indication of detraditionalization, but the extent to which this is actually a trend or merely a statistical fluctuation needs to be investigated by future studies. Regarding the expected gender differences, it appeared that, in the oldest cohorts, the relative mobility of women was somewhat higher than that of men. However, these differences were very small. In general, the relative mobility patterns of men and women appeared to be very similar. Again, these findings can be taken as an indication of detraditionalization, but it must be emphasized that the magnitude of the changes was small. As assumed in Hypothesis 3, relative and absolute mobility patterns of the genders were found to converge across cohorts.

When interpreting our results, it is important to keep in mind that we cannot draw causal conclusions. Rather, we provide a description of interrelationships in intergenerational mobility patterns. Our study demonstrates the potential of using administrative data for sociological research on intergenerational educational mobility. A limitation is that certain groups (older birth cohorts and individuals not born in Switzerland) are less likely to be linked in the administrative data, and are thus less likely to be included in our analysis sample. Therefore, we have only considered individuals born in Switzerland after 1950. Many studies of intergenerational mobility impose such a restriction by place of birth, for example to ensure that individuals have gone through the education system in the country of interest (for a paper analyzing intergenerational educational mobility for ethnic minorities, see Wanner, 2022). Regarding the age restriction, one might suspect some sort of life cycle bias, as individuals from the oldest birth cohorts and their parents were interviewed at a later point in the life course than those from the younger cohorts (interviewed 2011–2020). However, as we restricted our sample to individuals aged over 30 (by which age educational processes have largely been completed), we are convinced that our analyses do not suffer from such a bias. In general, our findings fit well with, and extend, existing research.

## Data availability statement

The datasets generated and analyzed for this study can be obtained from the FSO (Bundesamt für Statisitk) after signing a data use and linkage agreement. Further information can be found here: https://www.bfs.admin.ch/bfs/en/home/services/data-linkages/for-third-parties.html.

## Author contributions

RN and RB contributed to conception and design of the study and organized the database. RN performed the statistical analysis and wrote the first draft of the manuscript. RB wrote the second draft of the manuscript. Both authors contributed to manuscript revision and read and approved the submitted version.

## Footnotes

^1^Since we do not have data on occupational status or social class for a large part of our sample for which we have educational data, we cannot present classical OED analyses below.

^2^If an individual appeared multiple times in the data, we took the information given when that individual was closest to the age of 30.

^3^Previous research in Switzerland has shown that the educational choices of ethnic majority and ethnic minority students differ (e.g., Nennstiel, 2022a,b). For studies of the educational mobility of ethnic minorities in Switzerland that do not differentiate by birth cohort, see Bauer and Riphahn (2007) or Wanner (2022).

^4^The percentages from which the bar graphs shown below (Figures 2–4, 6–8) are derived are presented in tables in the Supplementary material (see Supplementary Tables 2–7 in the Supplementary material).

^5^Sankey diagrams are flow charts. The width of the arrows is proportional to the number of observations that constitute the flow from one categorical variable (e.g., parental education) to another categorical variable (e.g., type of educational mobility). Furthermore, the marginal distributions of the two categorical variables (proportional to the number of observations within the different categories of the variables) are also displayed at the sides of the graph.

^6^There are discussions in social mobility research about the extent to which mobility patterns can be estimated and interpreted when structures have changed fundamentally across generations, whether through occupational upgrading or through educational expansion (e.g., Seiler, 2018; Nennstiel, 2021). For a discussion of the extent to which measures of relative mobility may or may not need to be margin-free, see the discussion in Seiler (2018) and Seiler and Jann (2019).

^7^For our analyses, we utilized Stata17 by including some user-written ados (Jann, 2014, 2018; Naqvi, 2023), as well as the R Statistical Software (v4.1.0; R Core Team, 2021), by employing the DescTools package (Signorell, 2023).

^8^When considering the results, it is important to note that data on both parents are not available for each individual. For case numbers of the analysis sample by parental education, gender and cohort, see Supplementary Table 1 in the Supplementary material. For Figures 3–9, we computed the analyses again only for the individuals for whom educational information was available for both parents (see Supplementary Figures 5–11 in the Supplementary material). The results for this sample were almost identical to those presented here, so we have chosen to present the sample with the larger number of cases and the less rigorous selection criterion.

^9^In addition, we calculated Cramér's V and Goodman and Kruskal's gamma as measures of association for nominal and ordinal variables respectively (Agresti, 2002: 58f). We also calculated a summary measure of association based on entropy called the uncertainty coefficient (Agresti, 2002; Jann and Combet, 2012). This coefficient can take values between 0 and 1 and indicates the percentage by which the entropy in children's education is reduced when information about parents' education is taken into account. The time trends are very similar to those reported here (see Supplementary Figures 2–4 in the Supplementary material).
