# Supplementary material for: Gendered intergenerational educational mobility patterns converge in the cohort sequence: evidence from Switzerland using administrative data
Source: Front Sociol. 2023 May 9;8:1172553. doi: 10.3389/fsoc.2023.1172553 (PMC10203400; doi:10.3389/fsoc.2023.1172553)
Supplement: Supplementary file 1 [file Data_Sheet_1.pdf]

Supplementary Material

1 Supplementary Tables

	1951–1955	1956–1960	1961–1965	1966–1970	1971–1975	1976–1980	1981–1985	1986–1990	*Total*
**Own educational information**									
Total	21,403	50,150	88,892	107,328	98,723	94,012	74,296	21,308	556,112
Men	10,763	25,181	44,249	52,674	48,873	46,950	36,960	10,351	276,001
Women	10,640	24,969	44,643	54,654	49,850	47,062	37,336	10,957	280,111
**Fathers' educational information**									
Total	9,670	28,029	58,297	78,308	78,350	78,235	62,653	17,785	411,327
Men	5,005	14,250	29,128	38,590	38,991	39,142	31,212	8,642	204,960
Women	4,665	13,779	29,169	39,718	39,359	39,093	31,441	9,143	206,367
**Mothers' educational information**									
Total	18,169	42,269	75,392	91,940	85,663	82,510	65,631	18,489	480,063
Men	9,111	21,264	37,499	45,116	42,380	41260	32,752	9,033	238,415
Women	9,058	21,005	37,893	46,824	43,283	41250	32,879	9,456	241,648
**Both parents' educational information**									
Total	6,436	20,148	44,797	62,920	65,290	66,733	53,988	14,966	335,278
Men	3,353	10,333	22,378	31,032	32,498	33,452	27,004	7,324	167,374
Women	3,083	9,815	22,419	31,888	32,792	33,281	26,984	7,642	167,904

Supplementary Table 1. Case numbers of the analysis sample by own educational information, parental educational information and gender, across birth cohorts

Source: Structural survey cumulations 2011–2015 and 2016–2020, and STATPOP 2010–2020; unweighted, our own calculations

Highest educational attainment	1970	1980	1990	2000	2011–2015	2016–2020
Total						
No educational degree	0	1	1	2	5	4
Compulsory school	45	31	23	17	10	10
VET	36	44	51	45	30	28
General education	9	10	7	8	12	11
PET	5	8	11	14	15	16
University	4	7	8	12	28	33
Men						
No educational degree	0	1	1	2	5	3
Compulsory school	35	25	19	14	10	9
VET	44	46	50	43	30	29
General education	7	7	4	6	10	9
PET	7	12	16	20	18	17
University	6	9	10	14	28	32
Women						
No educational degree	0	1	1	2	5	4
Compulsory school	55	38	27	20	11	10
VET	28	41	52	47	29	27
General education	11	14	9	11	15	12
PET	3	4	6	9	13	14
University	1	4	5	10	27	34

Supplementary Table 2. Highest educational attainment of 30–39-year-olds in percentage points, separated by gender, across year of data collection

Source: Census 1970–2000, and structural survey cumulations 2011–2015 and 2016–2020; our own calculations

	1951–1955	1956–1960	1961–1965	1966–1970	1971–1975	1976–1980	1981–1985	1986–1990
**Highest parental educational attainment**								
No educational degree	19	15	12	9	6	4	3	2
Compulsory school	33	28	23	20	15	11	8	7
VET	32	36	39	41	43	43	42	43
General education	8	9	10	10	11	11	12	11
PET	6	8	11	14	16	19	21	22
University	3	4	5	6	9	12	14	15
**Father's education**								
No educational degree	14	12	10	8	6	5	4	3
Compulsory school	23	19	16	15	12	10	8	7
VET	40	43	44	45	45	45	45	46
General education	8	8	8	8	7	7	7	7
PET	10	13	15	17	18	20	22	22
University	5	6	7	8	10	12	14	14
**Mother's education**								
No educational degree	23	20	17	13	10	7	5	4
Compulsory school	40	37	34	31	26	21	17	14
VET	27	31	35	39	43	47	49	51
General education	8	8	9	10	12	13	14	13
PET	2	3	3	4	5	7	9	10
University	1	1	1	2	3	5	6	8

Supplementary Table 3. Highest parental educational attainment in percentage points, separated by parental education and birth cohort

Source: Structural survey cumulations 2011–2015 and 2016–2020, and STATPOP 2010–2020; unweighted, our own calculations

Total	1951–1955	1956–1960	1961–1965	1966–1970	1971–1975	1976–1980	1981–1985	1986–1990
Highest parental education								
Downward mobility	10	12	14	16	17	19	19	21
Lateral mobility	24	26	29	31	33	34	36	37
Upward mobility	65	62	57	53	50	47	45	42
Father's education								
Downward mobility	14	15	16	17	17	17	17	19
Lateral mobility	30	31	32	34	35	35	36	36
Upward mobility	56	54	51	49	48	48	47	45
Mother's education								
Downward mobility	6	6	7	7	8	9	11	12
Lateral mobility	20	20	21	23	24	26	28	31
Upward mobility	74	74	72	70	68	65	62	58
**Men**								
Highest parental education								
Downward mobility	8	9	11	13	14	17	19	22
Lateral mobility	22	24	26	29	32	33	36	37
Upward mobility	70	67	63	58	54	50	45	41
Father's education								
Downward mobility	11	12	13	13	14	15	17	20
Lateral mobility	28	29	30	32	34	34	36	36
Upward mobility	61	60	57	55	53	51	47	44
Mother's education								
Downward mobility	5	5	6	6	7	9	11	13
Lateral mobility	16	17	18	20	21	24	27	30
Upward mobility	79	78	77	74	72	67	62	57
**Women**								
Highest parental education								
Downward mobility	13	15	17	18	20	20	20	20
Lateral mobility	27	29	31	33	35	35	36	37
Upward mobility	60	57	52	48	46	45	44	43

Father's education								
Downward mobility	18	19	20	20	20	20	18	18
Lateral mobility	31	33	34	36	36	35	35	36
Upward mobility	50	48	46	44	44	45	47	46
Mother's education								
Downward mobility	7	7	8	8	9	10	10	11
Lateral mobility	23	23	25	27	27	28	29	31
Upward mobility	70	69	67	65	64	63	61	58

Supplementary Table 4. Absolute mobility patterns in percentage points across birth cohorts, separated by gender and operationalization of parental education

Source: Structural survey cumulations 2011–2015 and 2016–2020, and STATPOP 2010–2020; unweighted, our own calculations

Highest parental education	1951–1955	1956–1960	1961–1965	1966–1970	1971–1975	1976–1980	1981–1985	1986–1990
Without degree (100%)								
Downward mobility	0	0	0	0	0	0	0	0
Lateral mobility	5	4	3	2	2	2	3	2
Upward mobility	95	96	97	98	98	98	97	98
Compulsory education (100%)								
Downward mobility	3	2	1	1	1	1	1	2
Lateral mobility	12	10	8	7	6	6	8	9
Upward mobility	86	88	90	91	93	93	90	89
VET (100%)								
Downward mobility	7	6	5	4	4	4	4	5
Lateral mobility	48	49	50	51	49	45	45	47
Upward mobility	45	45	45	44	47	50	51	48
General education (100%)								
Downward mobility	36	37	38	40	37	34	33	34
Lateral mobility	18	14	14	13	12	14	12	11
Upward mobility	46	49	48	48	50	52	55	55
PET (100%)								
Downward mobility	53	50	49	48	45	42	40	43
Lateral mobility	22	25	27	28	28	26	25	24
Upward mobility	25	26	24	24	28	32	34	33
University (100%)								
Downward mobility	44	47	47	46	42	39	36	38
Lateral mobility	56	53	53	54	58	61	64	62
Upward mobility	0	0	0	0	0	0	0	0

Supplementary Table 5. Absolute mobility patterns conditional on parental education (measured using highest parental education) in percentage points, by birth cohort

Note: Rounded values, which is why the sum within a category does not always add up to 100 percent

Source: Structural survey cumulations 2011–2015 and 2016–2020, and STATPOP 2010–2020; unweighted, our own calculations

	1951–1955	1956–1960	1961–1965	1966–1970	1971–1975	1976–1980	1981–1985	1986–1990
**Highest parental education**								
Without degree (100%)								
Downward mobility	0	0	0	0	0	0	0	0
Lateral mobility	4	3	2	2	2	3	2	2
Upward mobility	96	97	98	98	98	97	98	98
Compulsory education (100%)								
Downward mobility	2	1	1	1	1	1	2	2
Lateral mobility	7	6	6	6	5	6	8	10
Upward mobility	91	93	93	93	94	93	91	88
VET (100%)								
Downward mobility	5	4	4	4	3	4	4	5
Lateral mobility	45	44	44	45	43	42	45	48
Upward mobility	51	52	52	52	54	54	51	47
General education (100%)								
Downward mobility	31	32	34	34	32	33	33	33
Lateral mobility	11	9	9	8	8	10	9	9
Upward mobility	58	59	58	58	60	57	58	57
PET (100%)								
Downward mobility	41	38	37	36	34	35	37	42
Lateral mobility	28	32	34	35	35	29	28	27
Upward mobility	31	31	29	29	31	36	35	31
University (100%)								
Downward mobility	34	39	40	40	37	36	37	42
Lateral mobility	66	61	60	60	63	64	63	58
Upward mobility	0	0	0	0	0	0	0	0

Supplementary Table 6. Absolute mobility patterns conditional on parental education (measured using highest parental education) in percentage points, by birth cohort (men only)

Note: Rounded values, which is why the sum within a category does not always add up to 100 percent

Source: Structural survey cumulations 2011–2015 and 2016–2020, and STATPOP 2010–2020; unweighted, our own calculations

Highest parental education	1951–1955	1956–1960	1961–1965	1966–1970	1971–1975	1976–1980	1981–1985	1986–1990
Without degree (100%)								
Downward mobility	0	0	0	0	0	0	0	0
Lateral mobility	6	4	3	2	2	2	3	3
Upward mobility	94	96	97	98	98	98	97	97
Compulsory education (100%)								
Downward mobility	3	2	2	1	1	1	1	2
Lateral mobility	16	14	11	9	7	7	9	9
Upward mobility	80	84	87	90	92	92	90	90
VET (100%)								
Downward mobility	10	8	7	5	4	4	5	4
Lateral mobility	51	54	56	58	54	49	45	46
Upward mobility	39	38	37	37	41	47	50	49
General education (100%)								
Downward mobility	41	41	43	45	42	35	32	35
Lateral mobility	26	20	19	18	17	18	15	12
Upward mobility	33	39	38	38	41	47	52	53
PET (100%)								
Downward mobility	67	62	62	60	55	49	43	43
Lateral mobility	15	17	20	20	21	22	23	22
Upward mobility	18	20	18	20	24	29	34	36
University (100%)								
Downward mobility	55	55	55	52	46	41	34	34
Lateral mobility	45	45	45	48	54	59	66	66
Upward mobility	0	0	0	0	0	0	0	0

Supplementary Table 7. Absolute mobility patterns conditional on parental education (measured using highest parental education) in percentage points, by birth cohort (women only)

Note: Rounded values, which is why the sum within a category does not always add up to 100 percent

Source: Structural survey cumulations 2011–2015 and 2016–2020, and STATPOP 2010–2020; unweighted, our own calculations

2 Supplementary Figures

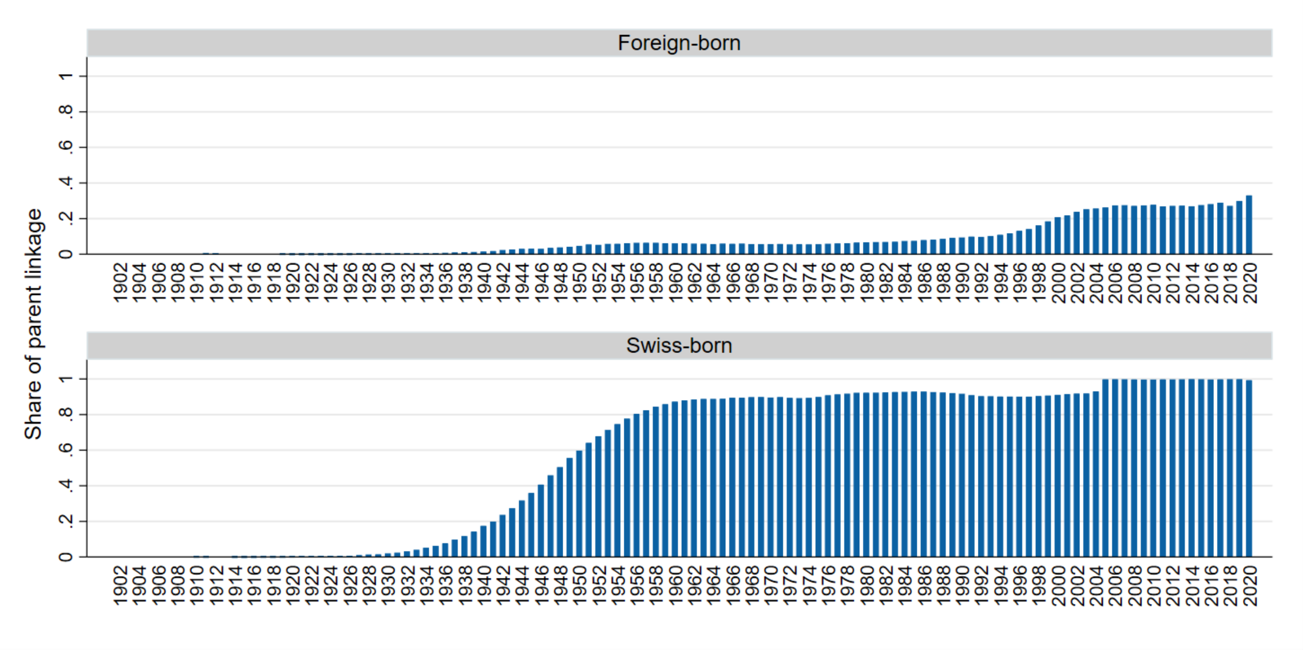

Supplementary Figure 1. Share of parental linkage (at least one parent) within STATPOP 2010–2020, differentiated by birth year and country of birth

Source: STATPOP 2010–2020; unweighted, our own calculations

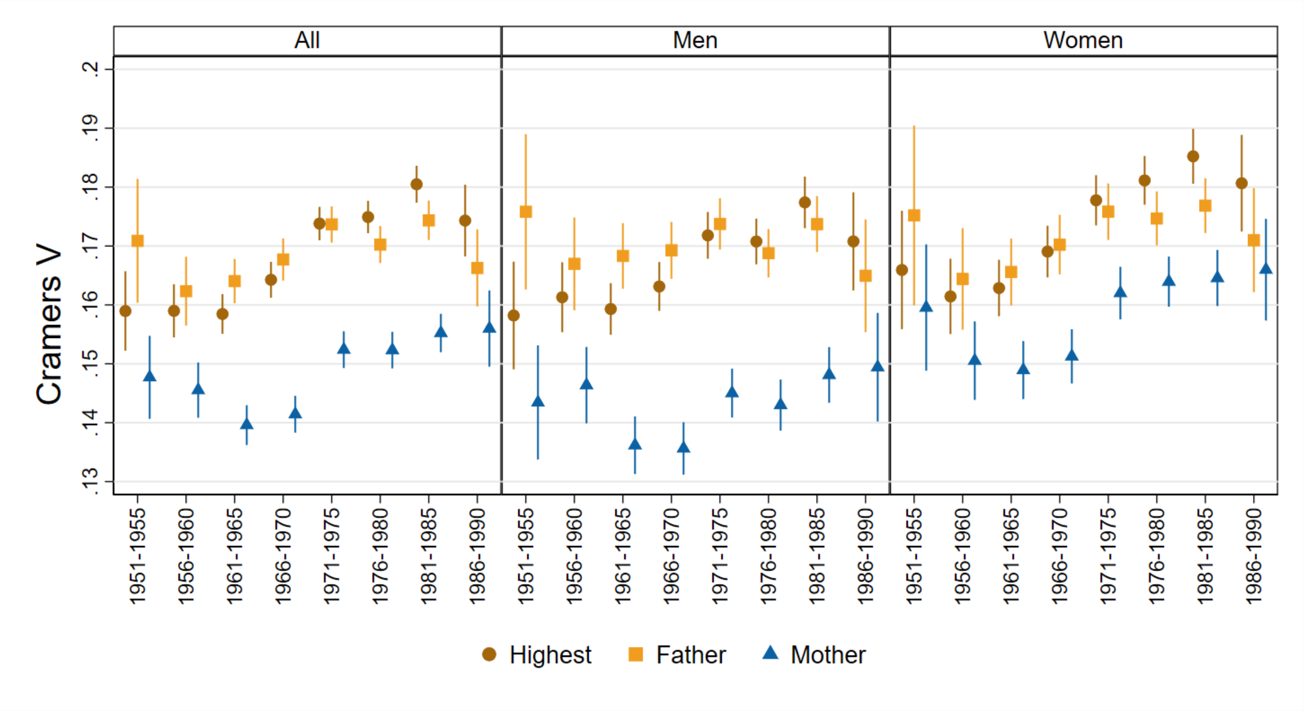

Supplementary Figure 2. Relative mobility patterns (Cramér's V) by gender across birth cohorts, differentiated by operationalization of parental education

Source: Structural survey cumulations 2011–2015 and 2016–2020, and STATPOP 2010–2020; unweighted, our own calculations

Note: Estimates based on 1,000 bootstrap replications

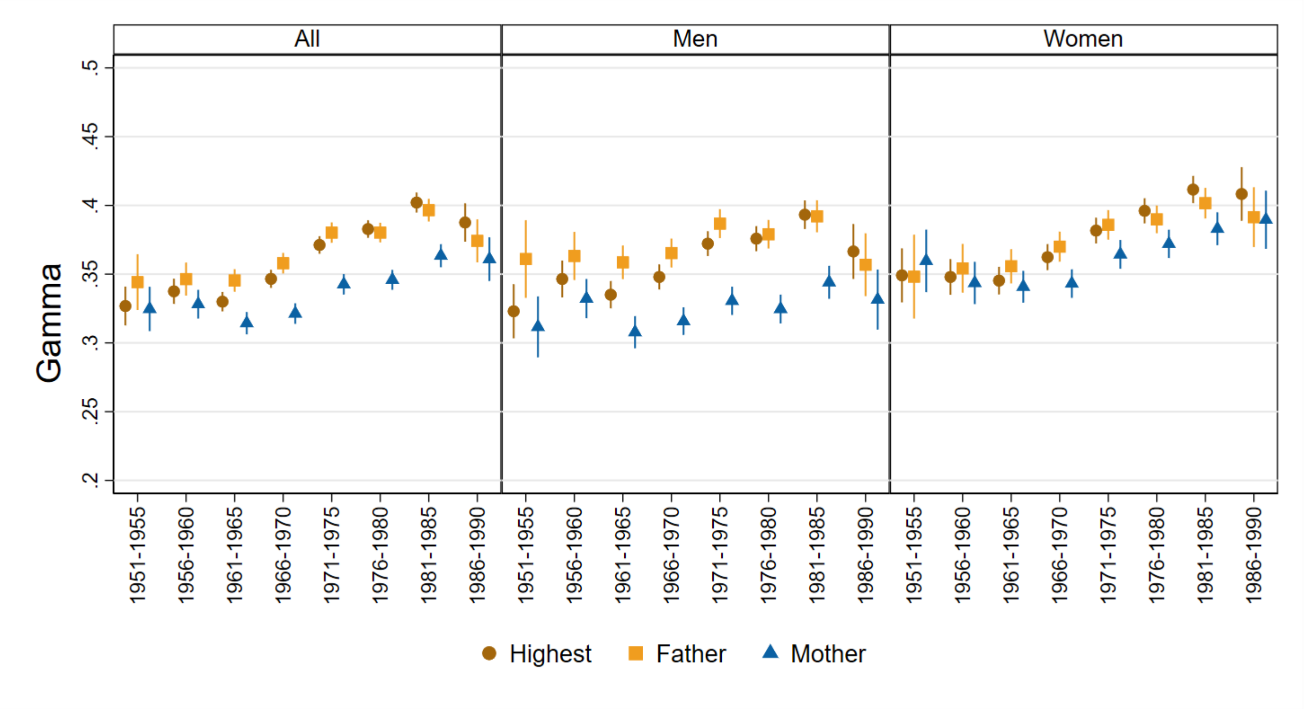

Supplementary Figure 3. Relative mobility patterns (Goodman and Kruskal's Gamma) by gender across birth cohorts, differentiated by operationalization of parental education

Source: Structural survey cumulations 2011–2015 and 2016–2020, and STATPOP 2010–2020; unweighted, our own calculations

Note: Estimates based on 1,000 bootstrap replications

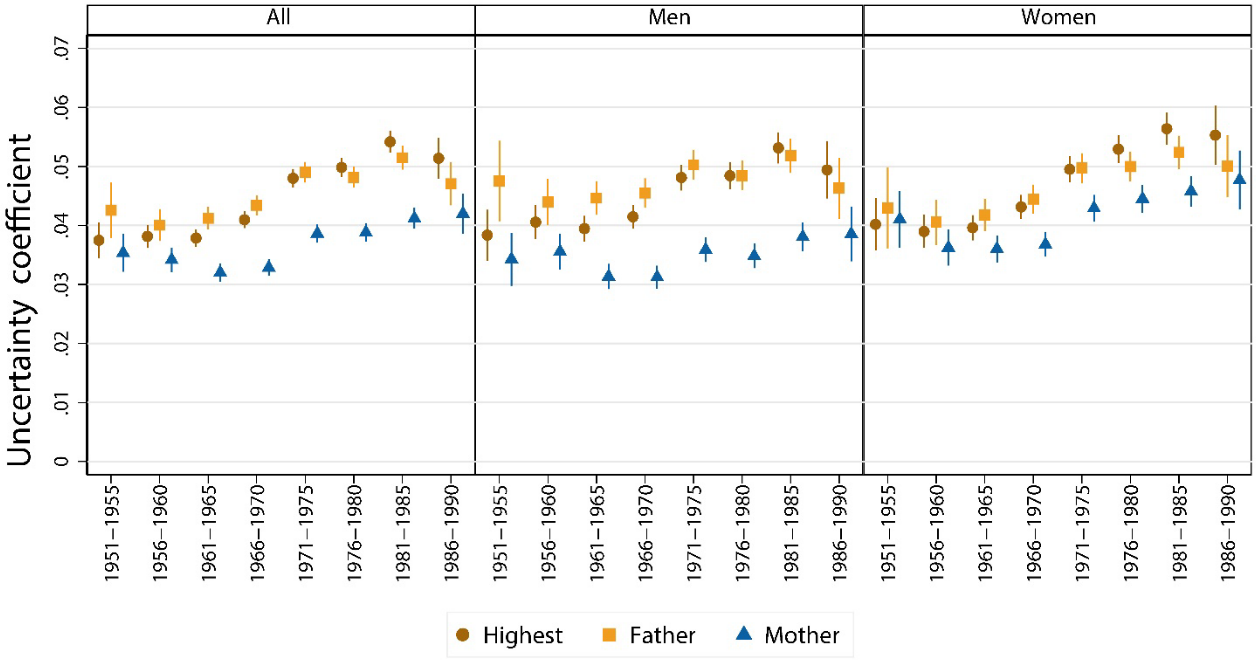

Supplementary Figure 4. Relative mobility patterns (uncertainty coefficient) by gender across birth cohorts, differentiated by operationalization of parental education

Source: Structural survey cumulations 2011–2015 and 2016–2020, and STATPOP 2010–2020; unweighted, our own calculations

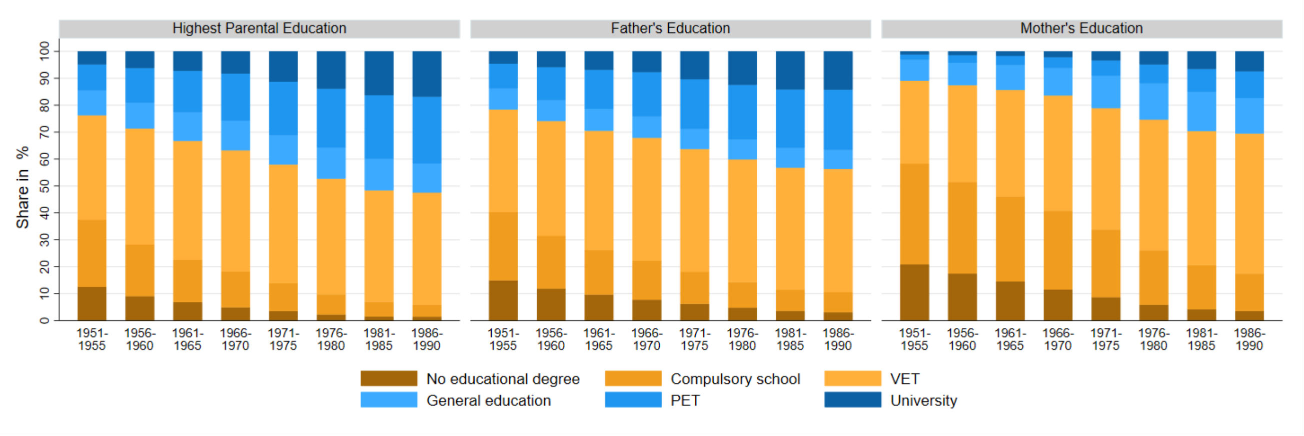

Supplementary Figure 5. Highest parental educational attainment, separated by parent and birth cohorts

Source: Reduced sample consisting only of persons for whom educational information is available for both parents

Source: Structural survey cumulations 2011–2015 and 2016–2020, and STATPOP 2010–2020; unweighted, our own calculations

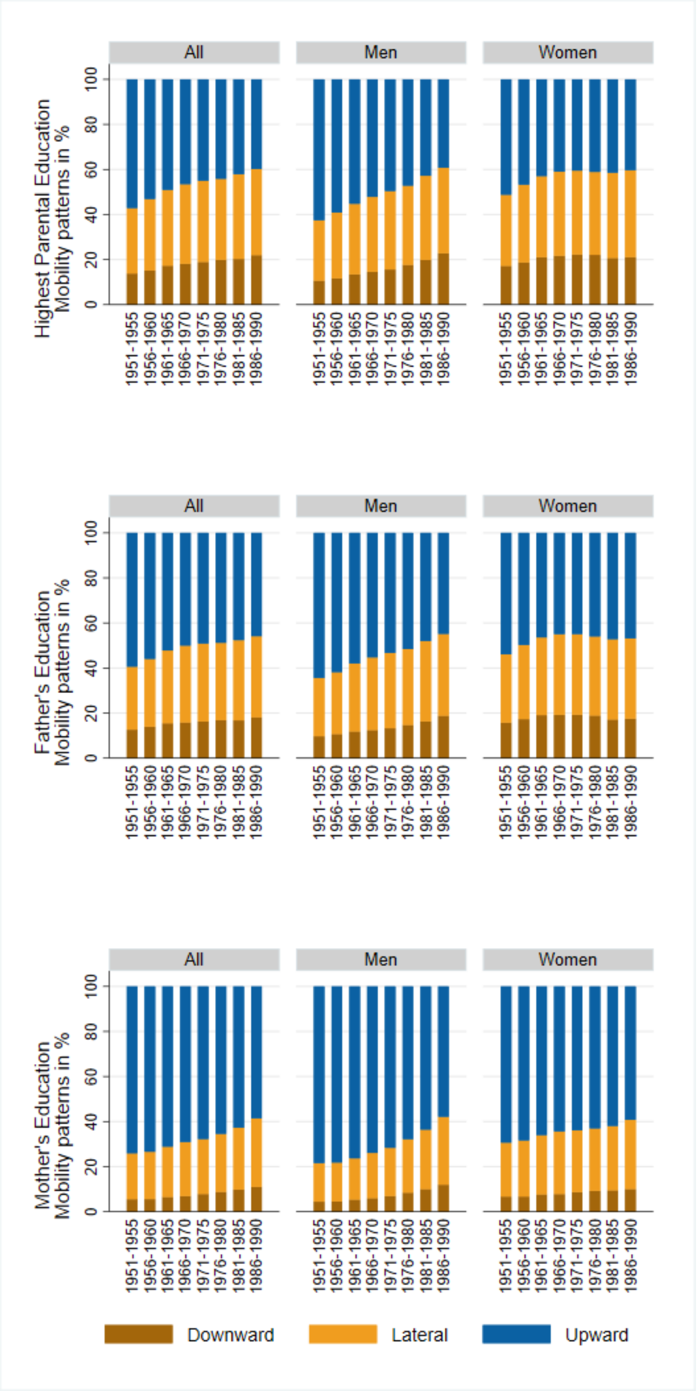

Supplementary Figure 6. Absolute mobility patterns across birth cohort, separated by gender and operationalization of parental education

Note: Reduced sample consisting only of persons for whom educational information is available for both parents)

Source: Structural survey cumulations 2011–2015 and 2016–2020, and STATPOP 2010–2020; unweighted, our own calculations

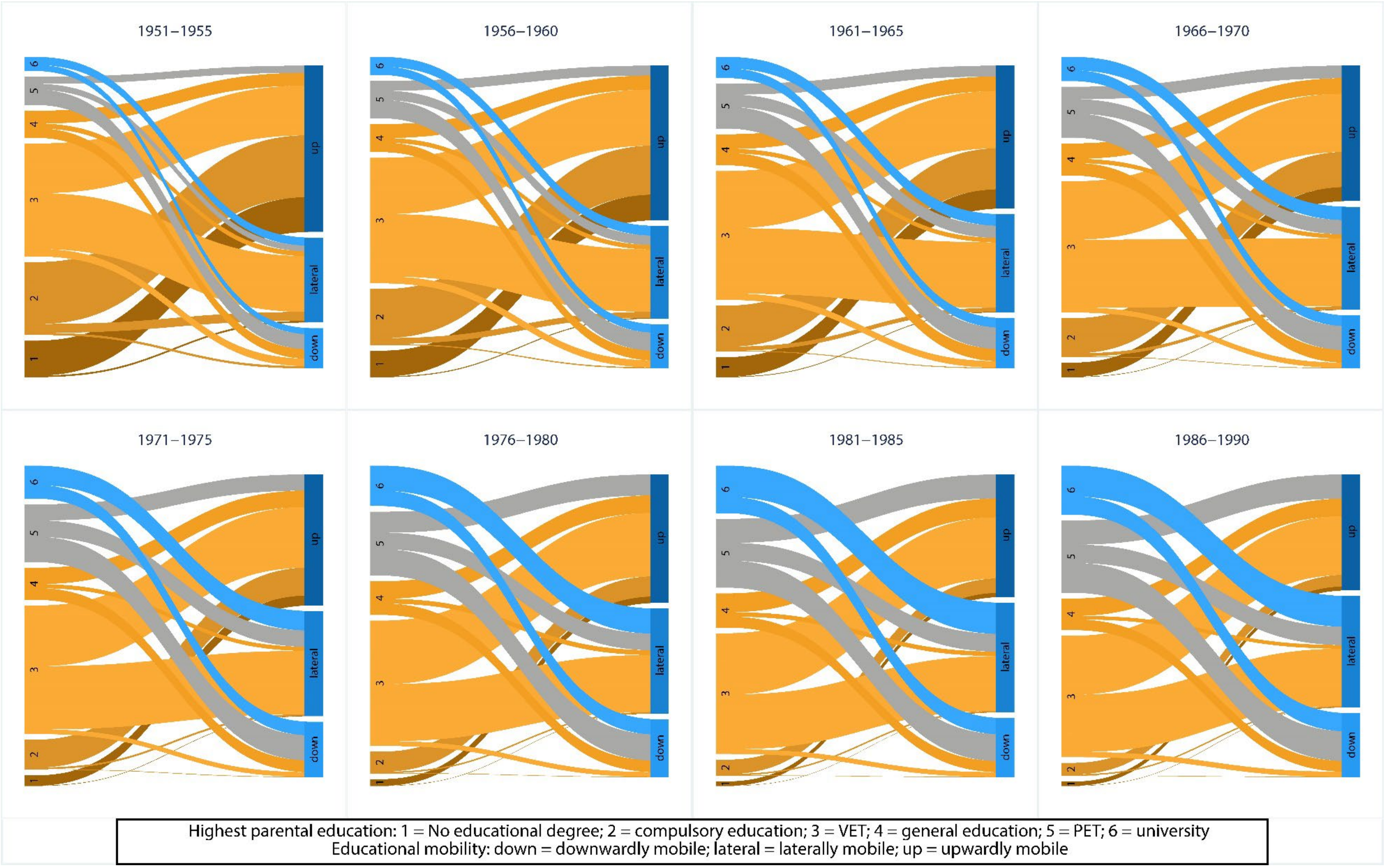

Supplementary Figure 7. Sankey plot of educational origin and educational absolute mobility (measured using highest parental education), by birth cohort
Note: Reduced sample consisting only of persons for whom educational information is available for both parents

Source: Structural survey cumulations 2011–2015 and 2016–2020, and STATPOP 2010–2020; unweighted, our own calculations

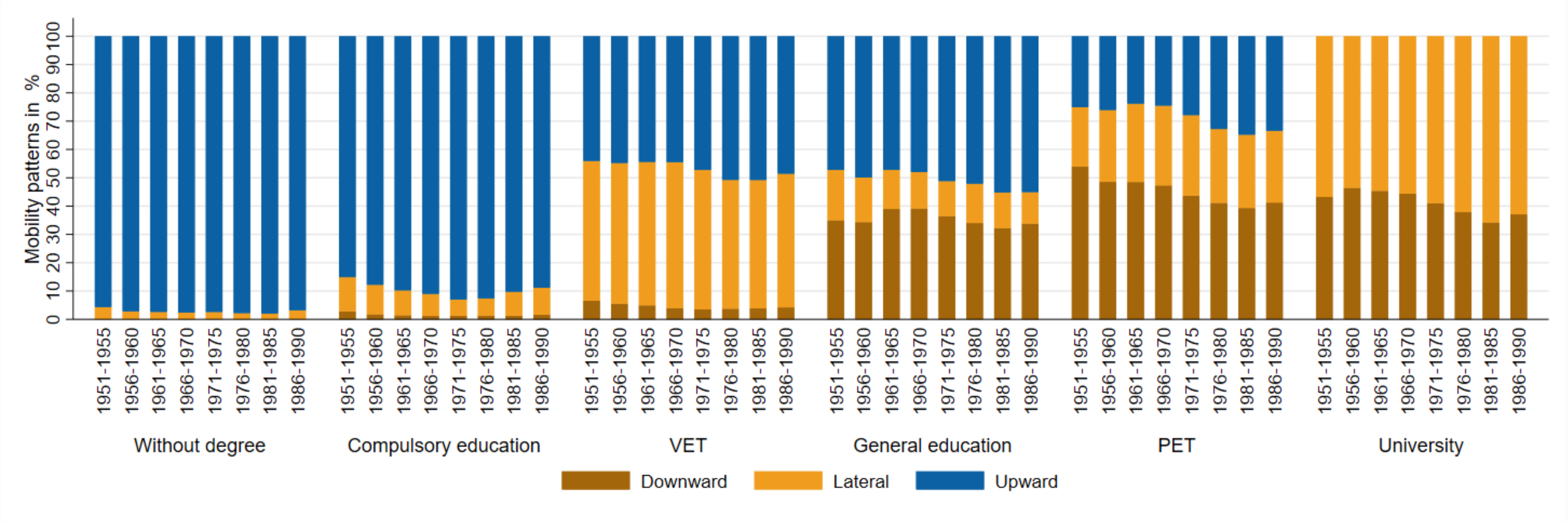

Supplementary Figure 8. Absolute mobility patterns conditional on parental education (measured using highest parental education), by birth cohort

Note: Reduced sample consisting only of persons for whom educational information is available for both parents

Source: Structural survey cumulations 2011–2015 and 2016–2020, and STATPOP 2010–2020; unweighted, our own calculations

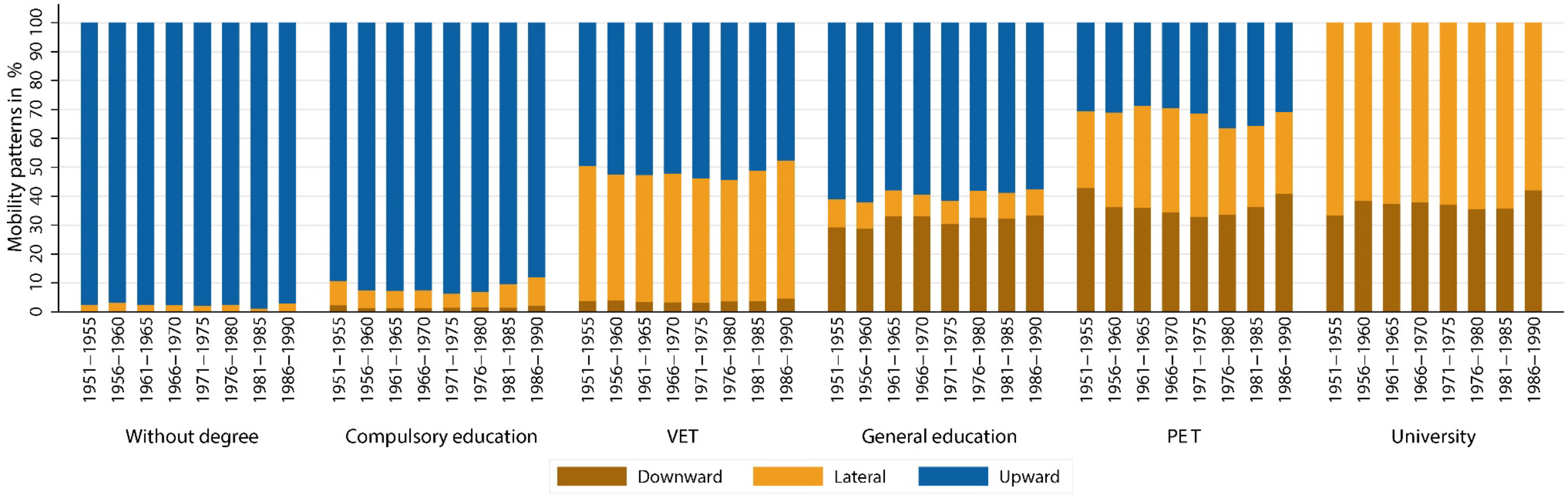

Supplementary Figure 9. Absolute mobility patterns conditional on parental education (measured using highest parental education), by birth cohort (men only)

Note: Reduced sample consisting only of persons for whom educational information is available for both parents

Source: Structural survey cumulations 2011–2015 and 2016–2020, and STATPOP 2010–2020; unweighted, our own calculations

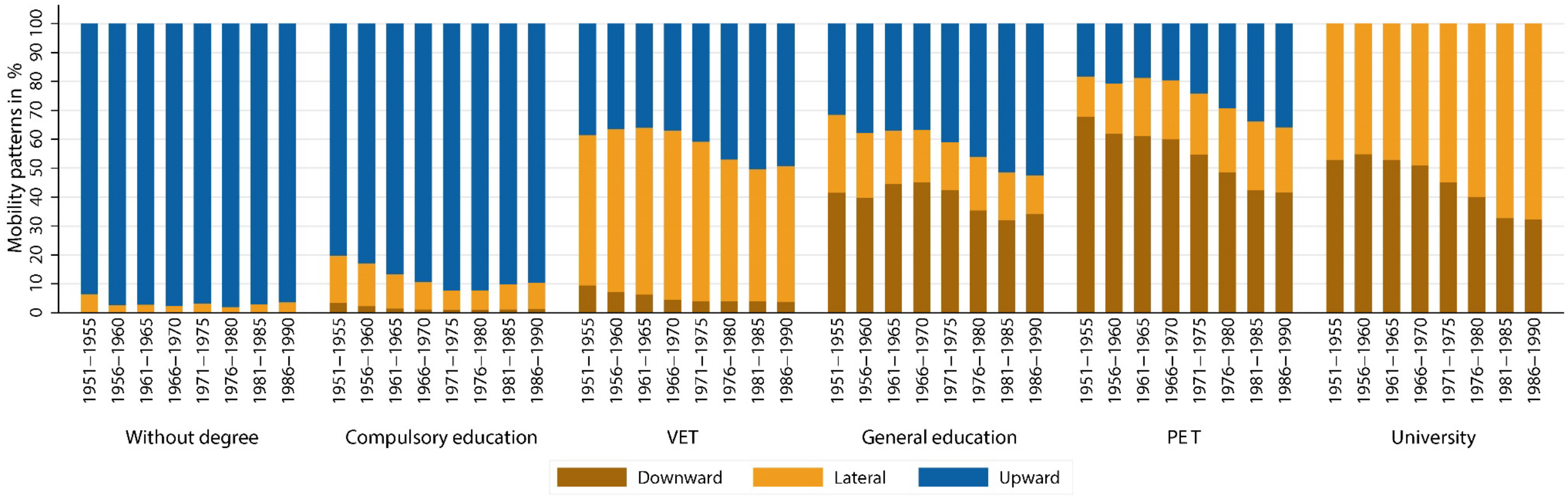

Supplementary Figure 10. Absolute mobility patterns conditional on parental education (measured using highest parental education), by birth cohort (women only)

Note: Reduced sample consisting only of persons for whom educational information is available for both parents

Source: Structural survey cumulations 2011–2015 and 2016–2020, and STATPOP 2010–2020; unweighted, our own calculations

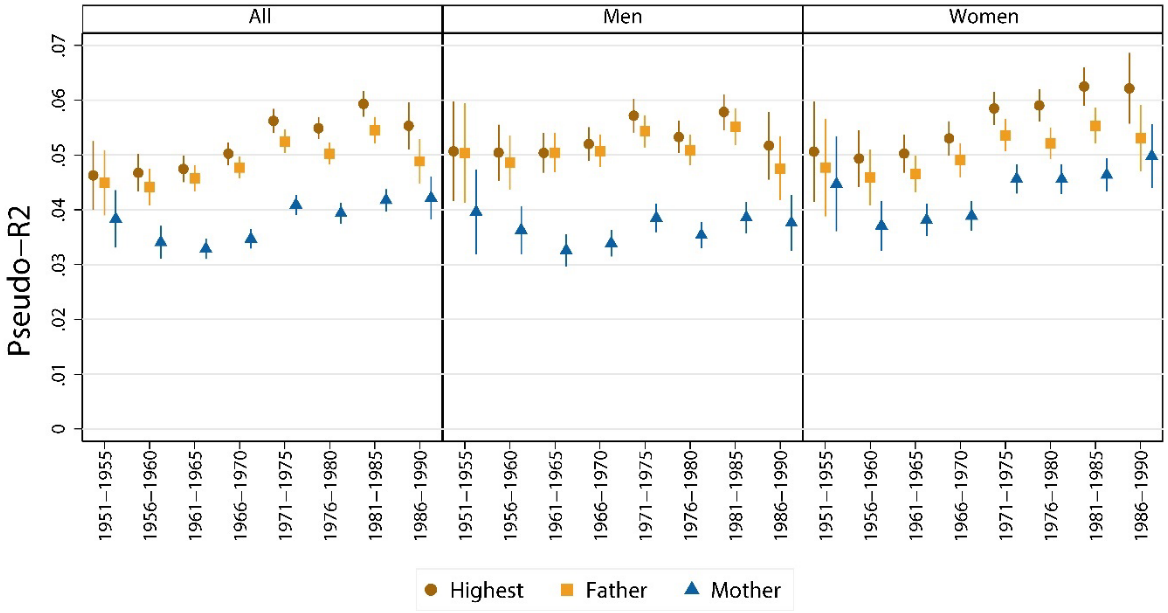

Supplementary Figure 11. Relative mobility patterns (pseudo-R^2) by gender across birth cohorts, differentiated by operationalization of parental education

Source: Structural survey cumulations 2011–2015 and 2016–2020, and STATPOP 2010–2020; unweighted, our own calculations

Note: Reduced sample consisting only of persons for whom educational information is available for both parents. Estimates based on 1,000 bootstrap replications of multinomial logit models
